# Acupuncture at KI3 in healthy volunteers induces specific cortical functional activity: an fMRI study

**DOI:** 10.1186/s12906-015-0881-3

**Published:** 2015-10-14

**Authors:** Bo Zhu, Yanjie Wang, Guifeng Zhang, Huailiang Ouyang, Jiping Zhang, Yu Zheng, Shaoqun Zhang, Chunxiao Wu, Shanshan Qu, Junqi Chen, Yong Huang, Chunzhi Tang

**Affiliations:** First Clinical School, Southern Medical University, Guangzhou, 510515 Guangdong Province China; School of Traditional Chinese Medicine, Southern Medical University, Guangzhou, 510515 Guangdong Province China; Zhaoqing Medical College, Zhaoqing, 526020 Guangdong Province China; Huarui Hospital, Southern Medical University, Guangzhou, 510630 Guangdong Province China; Clinical School of Acupuncture and Rehabilitation, Guangzhou University of Chinese Medicine, Guangzhou, 510405 Guangdong Province China

**Keywords:** Acupoint specificity, KI3, Sham acupuncture, Non-acupoint, Functional MRI

## Abstract

**Background:**

Using functional magnetic resonance imaging (fMRI), we determined brain regions that were activated/deactivated more by acupuncture at Taixi (KI3) than by non-acupoint or sham acupuncture.

**Methods:**

A total of 30 healthy volunteers were randomly divided into a KI3 group (15 subjects) and non-acupoint group (15 subjects). Subjects in KI3 group received a sham acupuncture and then a real acupuncture, fMRI was performed before and after sham acupuncture as well as after ture acupuncture. Subjects in non-acupoint group received a ture acupuncture and the fMRI was performed before and after ture acupuncture. The fMRI data obtained were successively analyzed using DPARSF2.3 and REST1.8 software, yielding regional homogeneity (ReHo) and amplitude of low frequency fluctuations (ALFF) values.

**Results:**

Compared with sham acupuncture, ALFF values were higher in Brodmann area (BA) 10 and lower in BA7 and BA18. ReHo values after real acupuncture at KI3 were higher in the right sub-lobar region and BA10 and were lower in BA31. Compared with the changes before and after real acupuncture at non-acupoint, the changes at KI3 showed higher ALFF valued in the left cerebellum posterior lobe, BA10, BA39, BA31 and decreased ALFF was observed in the BA18, BA19 and BA40; and higher ReHo values were shown in left cerebellum posterior lobe pyramis, left cerebellum anterior lobe. BA37, BA10, BA39, BA31 and lower ReHo values were shown in BA18 and BA31.

**Conclusion:**

Acupuncture at KI3 has a specific effect on certain brain regions associated with perception, body movement, spirit, and association. Additionally, visual and auditory cortices were affected, which may be related to the clinical applications of KI3 acupuncture in auditory and cognitive disorders, hypomnesis, loss of concentration, and the loss of ability to work and learn.

**Trial registration:**

The research ethics committee was achieved at 01/08/2012, the NO. was ChiECRCT-2012011. Website for Clinical Trial Registration: http://www.chictr.org.cn/showproj.aspx?proj=7123.

This study was registered at www.chictr.org, the Clinical Trial Registration Number was ChiCTR-TRC-12002427, and the registration number was achieved at 18/08/2012. The name of IRB that provided approval for the study and clearly state is *Chinese Clinical Trail Registry*.

## Background

Needling at acupoints has been applied clinically in traditional acupuncture for more than 2000 years. Li et al. [1] investigated the propagated sensation along meridians (PSM) produced by acupuncture at Quchi (LI11), a non-acupoint on meridian (control meridian point), and neither meridian nor acupoint (control point). PSM rate of the brachioradialis were measured using surface electromyography (amplitude and duration), showing that the PSM rate of LI11 (59.21 %) and the control meridian point (53.95 %) were significantly higher than the control point and the amplitude of LI11 was significantly higher than both the control meridian point and the control point. Zhang et al. [2] also reported that blood perfusion rate in the calf around the bladder meridian area was significantly higher after needling at the bladder-meridian acupoint than at a non-acupoint off the meridian. Hsiu et al. [3] stimulated the Hegu (LI4) and two nearby non-acupoints and simultaneously recorded the rate of blood-flow in the skin by Laser Doppler flowmetry and found that needling at LI4 significantly increased blood flow compared with needling at the non-acupoints. However, Linde et al. [4] concluded that needling at true acupoints did not produce obviously different results compared with needling at non-acupoints in the treatment of migraine. Thus, determining whether needling at true acupoints produces different results than at non-acupoints, and detecting whether acupoints have specificity is important.

Clinical observations have also produced relevant findings [5–10]. Wang et al. [5] evaluated the effectiveness of transcutaneous electrical acupoint stimulation (TEAS) at the Neiguan (PC6) in preventing postoperative nausea and vomiting in patients undergoing supratentorial craniotomy, and found that these symptoms were significantly lower after TEAS at the PC6 acupoint than at a non-acupoint. Ma et al. [6] observed effects of syndrome-differentiation acupuncture at true acupoints/non-acupoints on life quality in patients with functional dyspepsia, showing that the total effective rate, the SF-36, NDI and symptom total score in syndrome-differentiation acupuncture group were higher than non-acupoint group. Song et al. [7] compared the cumulative analgesic effect of EA stimulation of Sanyinjiao (SP6), Xuanzhong (GB 39) and non-acupoint for primary dysmenorrhea patients, Chen et al. [8] observe the clinical efficacy on sleep disorder in the intervention of flying needling therapy/non-acupoint acupuncture, showing that the total effective rate in the flying needling group was superior than non-acupoint acupuncture group, as the score of each item and the total score of PSQI in the flying needling group lower than non-acupoint acupuncture group. However, Shi et al. [9] compared visual analogue scale scores of patients with primary dysmenorrhea after acupuncture at Sanyinjiao (SP6), Xuanzhong (GB39), or a non-acupoint, and found no significant differences across treatments. Additionally, no statistically significant differences were observed in plasma PGE(2), PGF(2a), TXB(2), or 6-keto PGF(1a) levels. Using a rat model of adjuvant-induced arthritis, Li et al. [10] explored the efficacy and safety of acupuncture for post-stroke depression. The observation group received acupuncture at Baihui (GV20), Yintang (EX-HN3), Sishencong (EX-HN1), Taichong (LR3), etc. and the control group received acupuncture at non-acupoints, showing that the total effective rate between two groups was equivalent efficacy.

Experimental research has also compared the effects of acupuncture at true and non-acupoints [11–15]. Gao et al. [11] investigated cell proliferation and differentiation in the hippocampus of young rats that received electroacupuncture (EA) at bilateral LI11, Waiguan (TE5), Huantiao (GB30), Zusanli (ST36), or a non-acupoint. BrdU double labeling showed that greater cell proliferation, cell survival, and numbers of newly differentiated neurons occurred in the true EA group. Using a rat model of adjuvant-induced arthritis, He et al. [12] reported that EA at acupoints ST36, Xuanzhong (GB39), and Shenshu (BL23) markedly decreased paw swelling, the histologic scores of inflammation in the synovial tissue, and body weight loss. Further, immunostaining revealed greater levels of vasoactive intestinal peptide in the synovial tissue. In contrast, electrical stimulation at a non-acupoint did not yield these beneficial effects. Huang et al. [13] investigated genome-wide gene expressions and the cardioprotective effects of EA pretreatment at the PC6 on myocardial ischemia reperfusion (I/R) injury, finding that EA and electro-acupuncture at non-acupoint reversed some of these gene expression levels, and these genes were involved in multiple pathways, including ECM, MAPK signaling, apoptosis, cytokine and leukocyte pathways, while some pathways were uniquely regulated by EA, but not non-acupoint pretreatment, such as oxidative stress, cardiac muscle contraction, gap junction, vascular smooth muscle contraction, hypertrophic, NOD-like receptor, and P53 and B-cell receptor pathways. However, Bing et al. [14] found that Acupuncture, either applied at “Zusanli” or at a non-acupoint and noxious thermal stimulation induced similar strong inhibitory effects on the C-fibre-evoked responses of trigeminal convergent neurons. Zhang X et al. [15] evaluated the effect of acupuncture on the changes in the histomorphometric and mechanical properties of femurs in senescence-accelerated mice strain P6, showing that both acupuncture at Shenshu (BL23) and at non-acupionts significantly improved the scores for ultimate force, yield force, elastic stress, ultimate stress and energy to yield force.

In addition to comparing the effects of acupuncture at true and non-acupoints, investigators have also compared real and sham acupuncture. Vickers et al. [16] reported that real acupuncture was associated with improved pain outcomes compared with sham-acupuncture and no-acupuncture controls, with response rates of approximately 30 % for no acupuncture, 42.5 % for sham acupuncture, and 50 % for real acupuncture. Chou et al. [17] investigated the changes in blood flow/perfusion in the liver and spleen resulting from the application of 2 Hz EA, showing that sham EA did not increase the mean blood flow/perfusion in the liver or spleen; 2 Hz EA at bilateral Yinlingquan (SP9) acupoints increased the mean blood flow/perfusion in the spleen, but not in the liver. In contrast, 2 Hz EA at bilateral Ququan (LR8) acupoint increased the mean blood flow/perfusion in the liver, but not in the spleen. However, Kapatkin et al. [18] observed the effects of electrostimulated acupuncture on ground reaction forces and pain scores in dogs with chronic elbow joint arthritis, finding there did not have a significant effect on ground reaction forces for any limb between acupuncture and sham acupuncture. Mao et al. [19] reported that both electroacupuncture and sham acupuncture improved arthralgia related to aromatase inhibitors in breast cancer patients.

The specificity of acupoint stimulation is thus a focus of acupuncture research, and usually includes comparisons between non-acupoint or sham acupuncture. Further, results appear to be contradictory, showing that a consensus regarding the efficacy of acupuncture has yet to be reached. Xing et al. [20] reviewed acupoint specificity and indicated that point specificity does exist in acupuncture, but that sham acupoints and the placebo phenomenon need to be seriously considered.

Recently, functional magnetic resonance imaging (fMRI) has been used in acupoint-specificity research. Zhong C et al. [21] assumed the relatively functional specificity of acupoints may evolve as the function of time, and explored the causal interactions within and among the post-acupuncture resting-state networks (RSNs) at a hearing-related acupoint GB40, or the cognition-related acupoint KI3, finding acupuncture at different acupoints may exert different evolutive effects on causal interactions within and across the RSNs during segmented post-stimulus resting states.and Feng et al. [22] investigated the functional correlations throughout the entire brain following acupuncture at Zusanli (ST36)/non-acupoint, finding that increased correlations for acupuncture at ST36 compared to non-acupoint were primarily related with the limbic/paralimbic and subcortical regions such as the insula, amygdala, anterior cingulate gyrus, and thalamus, whereas decreased correlations were mainly related with the sensory and frontal cortex. Huang et al. [23] investigated the differences in activated/deactivated cortical regions after real/sham acupuncture at Waiguan (SJ5) or acupuncture at non-acupoint, demonstrating that compared with sham needling, real needling at SJ5 activated the BA8 area and left cerebellum. Compared with needling at the non-acupoint, needling at SJ5 activated the BA2 area, the left cerebellum and the right inferior semilunar lobule.

Analysis of regional homogeneity (ReHo) and amplitude of low frequency fluctuations (ALFF) are common methods for measuring cortical function. ReHo is one of the quantitative properties of resting-state fMRI that characterizes the similarity of local brain activity across a region [24]. ReHo primarily reflects the synchronism of a time series in regional brain areas, not signal intensity, and it indirectly reflects the synchronism of the spontaneous activity of local neurons in a specific brain region [25]. This has been shown to advance the understanding of the complexity of brain function. Another primary quantitative property of the resting-state BOLD signal is the amplitude of ALFF, which measures the total power within the range of 0.01 and 0.1Hz [26]. ALFF is based on the amplitude or strength of the regional brain activity and represents the intensity of a blood oxygen level–dependent signal in each voxel, which directly reflects the spontaneous activity of neurons via energy expenditure [27]. A study found that the reduced low-frequency fluctuations in white matter relative to gray matter by approximately 60 % suggest that ALFF is associated with field potential activity in local brain regions [28]. Therefore, ReHo and ALFF analyze different aspects of fMRI data, which leads to some differences in findings. Because the two analyses can find common changes in cortical functional regions, both were adopted to reduce inaccuracies and to provide reliable and comprehensive conclusions.

Because proof of acupoint specificity needs firm evidence, we chose a commonly used acupoint KI3 for the investigation, and compared fMRI data after acupuncture at KI3 with those after non-acupoint and sham acupuncture. We hypothesize that KI3 acupuncture, its clinical applications, and the resulting changes in functional brain activity are related.

## Methods

### Study design

This is a random, single-blind trial in which all volunteers were blind to the interventions they received.

### The setting

The study was performed at The First Affiliated Hospital of Guangzhou University of Chinese Medicine.

### Participants

The recruit of healthy volunteers first started at 05/09/2012, and the first scan of healthy volunteers was performed at 14/10/2012. Thirty healthy college-student volunteers in Guangzhou, PRC participated in the study. They were: (1) aged 21–28 years, (2) had never had acupuncture, (3) were eating well and not addicted to tobacco, tea, or coffee, (4) slept normally (before 0:00 am), (5) were moderate in size (BMI between 19 and 25), and (6) had no history of mental or nervous system diseases. Additionally, they (7) had had no abnormal pain (including dysmenorrhea) or insomnia within in the month preceding the study, (8) no metal implants such as heart stents or metal teeth, (9) no skin diseases or skin damage at the acupoint, (10) did not have thrombocytopenia, hemophilia, or other blood coagulation-dysfunction diseases, and (11) were not claustrophobic. Further, 1 month before testing, we screened out volunteers who were predicted to have no response or oversensitive responses to acupuncture through the acupuncture-response prediction test.

**Fig. 1:**
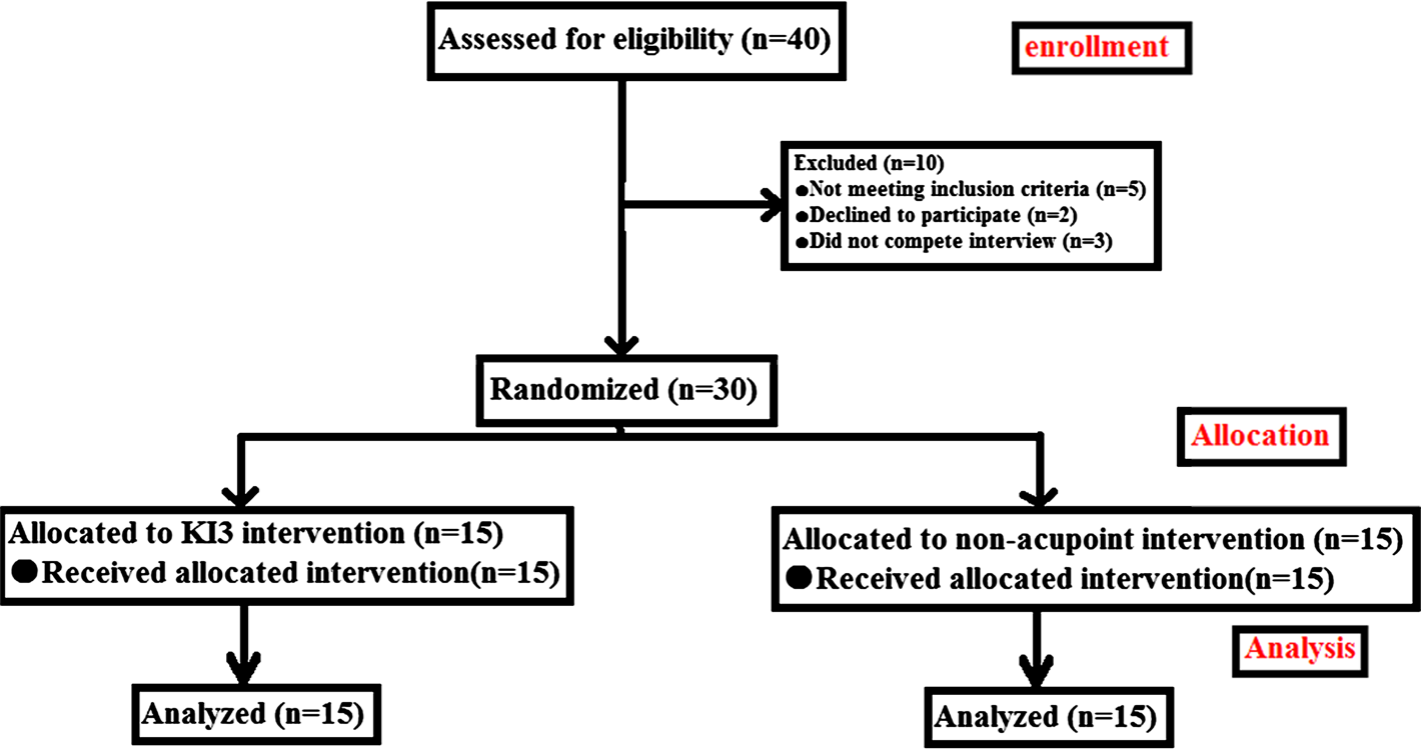
Consort flowchart the flow chart of this study according to the consort statement

This clinical trial was registered in China and approved by the ethical review committee (ChiECRCT-2012011) and clinical trial registry in China (ChiCTR-TRC-12002427). All the volunteers must sign the appropriate informed consent paperwork to indicate that they consent to participate (Fig. 1).

### Grouping method

The 30 volunteers were randomly divided into KI3 and non-acupoint groups (15 participants each) according to a random-numbers table and random-numbers was in a sealed envelope. Sham acupuncture consisted of needles that did not pierce the skin at K13. There was no significant difference in age, gender, height, and weight between the 2 groups.

### Experimental procedure

**Fig. 2:**
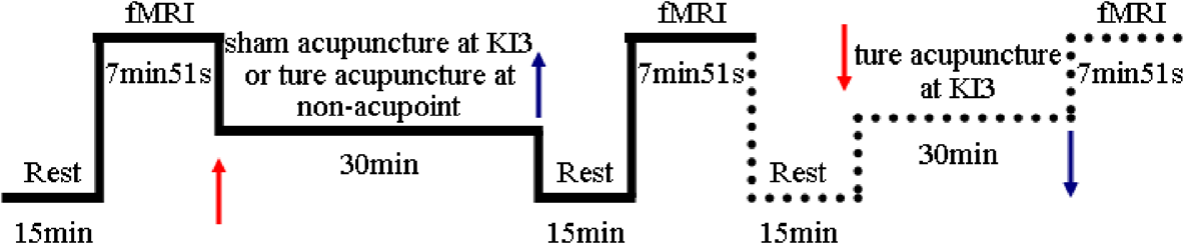
Trail Flow Chart. The subjects in KI3 group received two acupuncture (sham first and then a true acupuncture) and three fMRI, while subjects in non-acupoint group received only ture acupuncture and two fMRI, so the second acupuncture and third fMRI in KI3 group are showed by dotted line. Red arrow represents needle insertion, blue arrow represents needle withdrawal

All subjects both in KI3 group and in non-acupoint group took a 15-min rest at the beginning of the experiment and then each participant was given a baseline fMRI scan. Next, participants in the KI3 group received sham acupuncture for 30 min, after withdrawing the needle and resting for 15 min, participants received the second fMRI scan. Participants rested for 15 min followed by the real acupuncture, then the third fMRI scan was performed after a 15 min-rest rest followed needle withdrawal. While subjects in non-acupoint group received real acupuncture after baseline fMRI scan. The second fMRI scan was performed after the real acupuncture and a 15-min rest. The timeline of acupuncture stimulation and MRI scan is shown in Fig. 2.

### Acupuncture program

#### Acupuncture point location

**Fig. 3:**
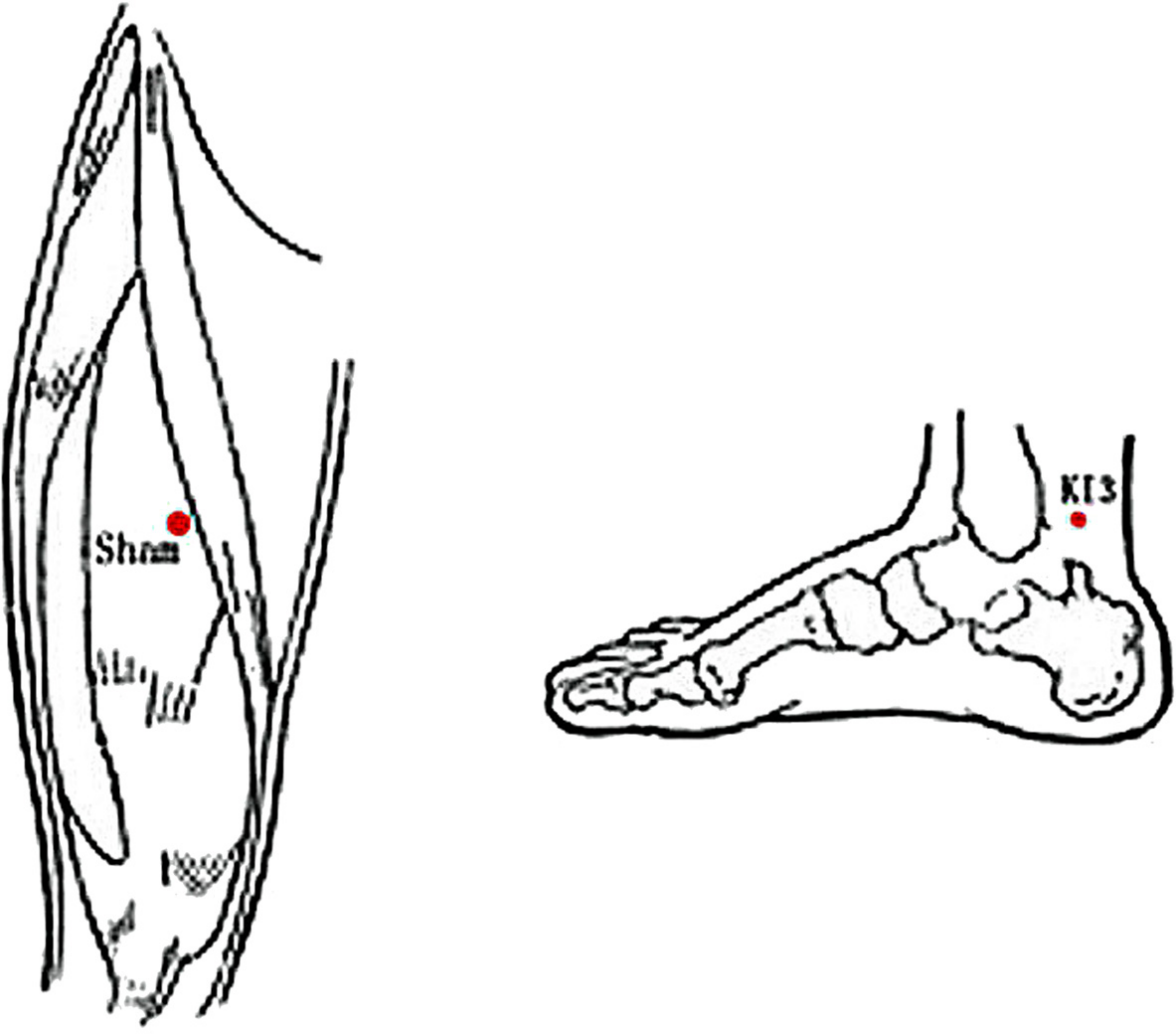
KI3 and Sham point locations

According to the national standards of the Location of Points (GB12346-90) [29], KI3 is located in the medial part of the foot at the cavity between the rear medial malleolus and the tendon of heel bone and the non-acupoint was located on the thighs, 2 cm medial to the midpoint of the line attaching the anterior superior iliac spine and the bottom-outer corner of the patellar tendon (Fig. 3).

#### Acupuncture procedure

Acupuncture needles (0.30 × 25–45 mm, HwaTo, Medical Supplies Co., Ltd. Suzhou) were inserted to 2 cm deep. After Deqi, twisting (90–180 °, 60–90 times/min), and lifting-thrusting (0.3–0.5 cm, 60–90 times/min) were performed for 1 min. This was repeated every 10 min for a total of 3 times over a 30-min span. Volunteers wore blindfolds during acupuncture.

### Scan procedure

Scanning was performed using a 3.0T Signa HDxt with an 8-channel head coil MRI scanner (GE Company, Fairfield, Connecticut, America). A T1-weighted sequence was administered for 1 min 51 s using an Fast Spin Echo sequence. Parameters for the OAx T1 FLAIR were: repetition time (TR) = 1750 ms, echo time (TE) = 24 ms, inversion time (IT) = 960 ms, field of vision (FOV) = 24 cm × 24 cm, image matrix = 320 × 224, number of excitations (NEX) = 1, layer thickness = 5.0 mm, interval = 1.0 mm, 30 layers total, echo train length = 8, bandwidth = 31.25.

Resting-state fMRI BOLD statistics were obtained by 6 min of scanning with a gradient-Echo Planar Imaging (GR-EPI) sequence, with these parameters: TR = 3000 ms, TE = 20 ms, flip angle (FA) = 90°, FOV = 240 mm × 240 mm, layer thickness = 5.0 mm, interval = 1.0 mm, 30 layers total, image matrix = 96 × 96, NEX = 1.

### Data analysis and statistical methods

#### Preprocessing

The initial resting-state fMRI statistics were preprocessed with Preprocessing was performed using Data Processing Assistant for Resting-State fMRI 2.3 (DPARSF. http://www.restfmri.net/forum/DPARSF) [30] which is based on Statistical Parametric Mapping (SPM8, http://www.fil.ion.ucl.ac.uk/spm) and Resting-State fMRI Data Analysis Toolkit (REST. http://www.restfmri.net) [31], including conversion to Digital Imaging and Communications in Medicine (DICOM) format, removing first 10 time point, slicing timing, realignment, spatial standardization, and spatial smoothing. Realignment calculated the translation on the X, Y, and Z axes and head motion during the scanning to exclude those with 3D translation of >1.5 mm or 3D rotation >1.5°. Spatial standardization fit images to the Montreal Neurological Institute (MNI) standard brain and we excluded those with low degrees of standardized fitting. Spatial smoothing was carried out with the 4 mm × 4 mm × 4 mm Gaussian kernel. After preprocessing, 30 subjects were included in statistical analyses.

#### ReHo analysis

After spatial standardization, linear regression was employed by DPARSF 2.3 software to remove the linear trends from the preprocessed data. Then nuisance covariates regression was performed, and covariates included head motion with ripid-body 6 head model, white matter signal, cerebrospinal fluid signal. Subsequently, we calculated the Kendall’s coefficient of concordance (KCC) value of each voxel to obtain an individual KCC map or ReHo map. These maps underwent whole-brain equalization for further statistical analysis [24, 25, 30].

#### ALFF analysis

After spatial smoothing, linear regression was employed by DPARSF 2.3 software to remove the linear trends from the preprocessed data. The extracted time curve was then convolved with a Hamming band-pass filter to obtain the low frequency oscillation signal amplitude (0.01–0.1 Hz). Subsequently, we calculated the ALFF value for the whole-brain voxels and obtained the ALFF map, which was then divided by the average ALFF value. This standardized ALFF map was then delineated [26, 27, 30].

#### Statistical analysis

Using REST 1.8 software finish the statistical analysis. The standardized ALFF and ReHo values of sham acupuncture at KI3 and real acupuncture at KI3 were calculated with paired *t*-test to find changes of ALFF and ReHo between real and sham acupuncture at KI3. The multiple comparison correction was performed by AlphaSim correction (Cluster Connectivity Criterion: edge connected; rmm = 5, continuous voxel >85). The sensation data was calculated as covariate during the paired *t*-test. Changes of ALFF and ReHo in KI3 group or non-acupoint group were determined by the contrast between baseline and after real acupuncture. Statistical ALFF and ReHo maps were constructed by computing a paired *t*-test between baseline and after real acupuncture ALFF and ReHo values (AlphaSim correction at *P* < 0.05, edge connected cluster connectivity criterion, rmm = 5, continuous voxel >85). Using REST1.8 software to calculate the ALFF and ReHo maps, then performed a direct comparison of the ALFF and ReHo changes between the KI3 and non-acupoint group. Then, using REST1.8 software mapped the location of voxels that had significantly different ReHo or ALFF values between conditions to MNI coordinate space.

## Results

Brain functional images of 30 cases were all included in the analysis, and there was no significant difference between groups in functional imaging before acupuncture.

### Changes of ALFF and ReHo values between real and sham acupuncture at KI3

#### ALFF analysis

Increased ALFF was detected in the Left cerebrum frontal lobe medial frontal gyrus (BA10). Decreased ALFF was observed in the right parietal lobe and precuneus (BA7), left occipital lobe lingual gyrus (BA18).

#### ReHo analysis

**Fig. 4:**
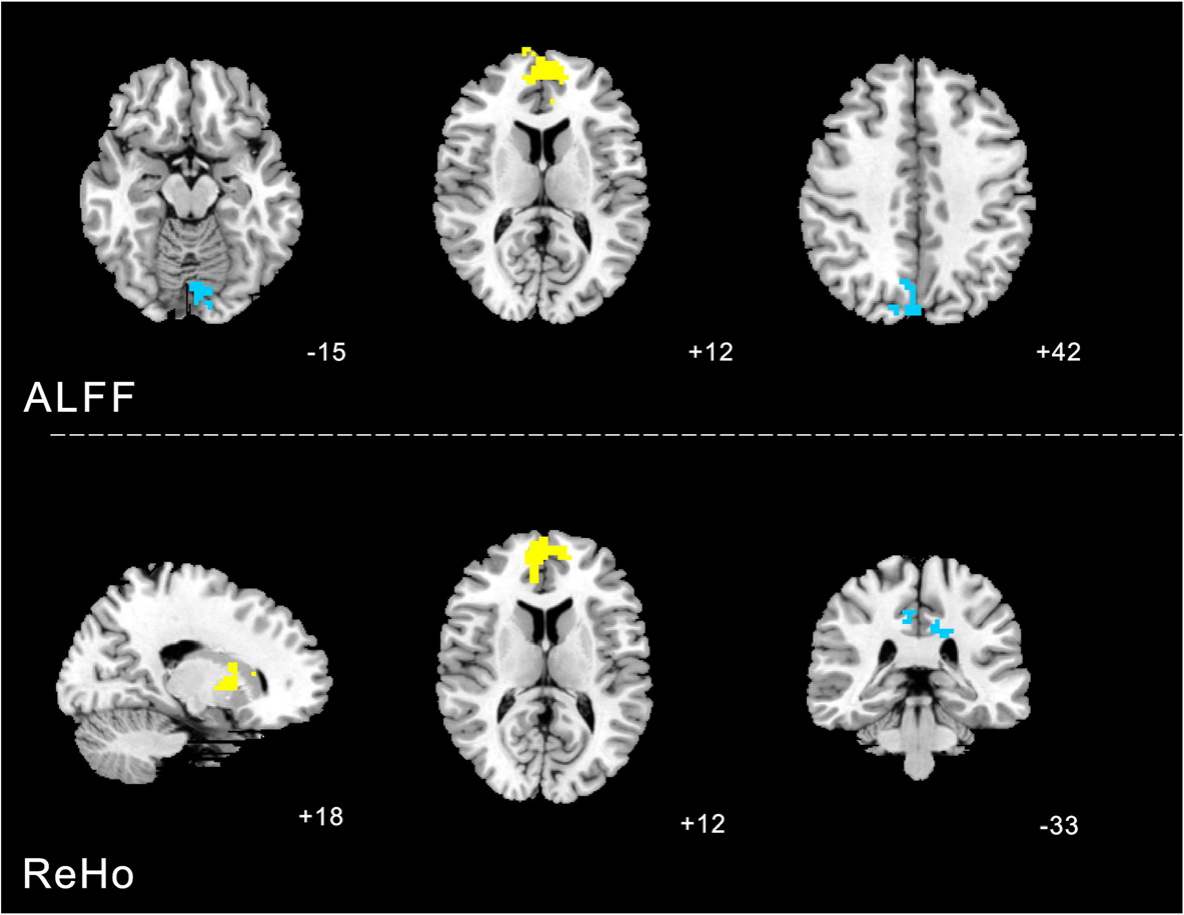
Changes of ALFF and ReHo values between real versus sham acupuncture at KI3 were calculated with paired *t*-test. The yellow color respects regions with higher ALFF and REHO values after real acupuncture at KI3 than sham acupuncture at KI3. The blue color respects regions with lower ALFF and REHO values after real acupuncture at KI3 than sham acupuncture at KI3

Increased ReHo (T value was positive) was detected in the right sub-lobar, right medial frontal lobe (BA10). Decreased ReHo (T value was negative) was observed in the left cerebrum limbic lobe (BA31) (Table 1, Fig. 4).

**Table 1:** Changes of ALFF and ReHo values between real versus sham acupuncture at KI3

Cluster	Voxels	Brain areas	Brodmann area (BA)	Peak MNI coordinate			Peak intensity
				X	Y	Z	
ALFF	112	Right cerebrum sub-lobar	-	18	0	0	4.3743
	305	Right frontal lobe frontal gyrus	10	6	54	12	6.5696
	87	Left limbic lobe	31	−12	−33	36	−4.104
REHO	91	Right parietal lobe and precuneus	7	3	−78	42	−3.3641
	331	Left medial frontal gyrus	10	−9	60	12	5.6462
	165	Left occipital lobe lingual gyrus	18	−3	−84	−15	−3.8456

### Changes of ALFF and ReHo values before and after acupuncture at KI3

#### ALFF analysis

Increased ALFF was detected in the left cerebellum posterior lobe and left middle frontal gyrus (BA10). There was no value decreased after real acupuncture.

#### ReHo analysis

**Fig. 5:**
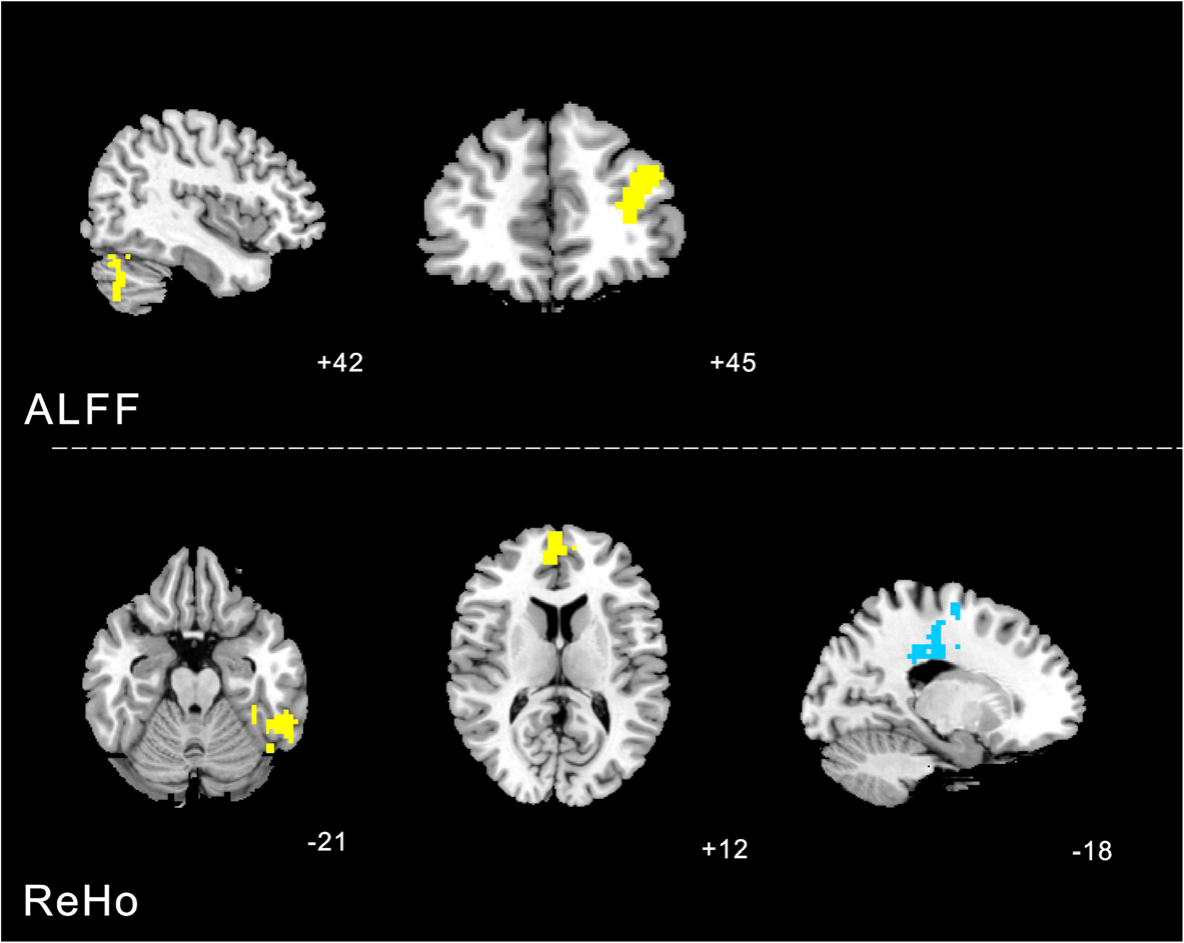
Changes of ALFF and REHO values before and after acupuncture at KI3 were calculated with paired *t*-test. The yellow color respects regions with higher ALFF and REHO values after real acupuncture at KI3 than before. The blue color respects in contrast

Increased ReHo was detected in the left temporal lobe fusiform gyrus (BA37) and right medial frontal gyrus (BA10). Decreased ReHo was observed in the left limbic lobe cingulate gyrus (BA6) (Table 2, Fig. 5).

**Table 2:** Changes of ALFF and REHO values before and after acupuncture at KI3

	Voxels	Brain areas	Brodmann area (BA)	Peak MNI coordinate			Peak intensity
				X	Y	Z	
ALFF	88	Left cerebellum posterior lobe	-	42	−72	−45	4.1072
	109	Left middle frontal gyrus	10	−33	45	21	4.437
REHO	134	Left temporal lobe fusiform gyrus	37	−45	−48	−21	4.7063
	136	Right medial frontal gyrus	10	6	54	12	6.0951
	152	Left limbic lobe cingulate gyrus	6	−18	−21	39	−7.4947

### Changes of ALFF and ReHo values before and after acupuncture at non-acupoint

#### ALFF analysis

Increased ALFF was detected in the left occipital lobe lingual gyrus (BA18), right middle occipital gyrus (BA19) and right parietal lobe postcentral gyrus (BA40). Decreased ALFF was observed in the right superior frontal gyrus (BA10), right middle temporal gyrus (BA39) and right parietal lobe and limbic (BA30).

#### ReHo analysis

**Fig. 6:**
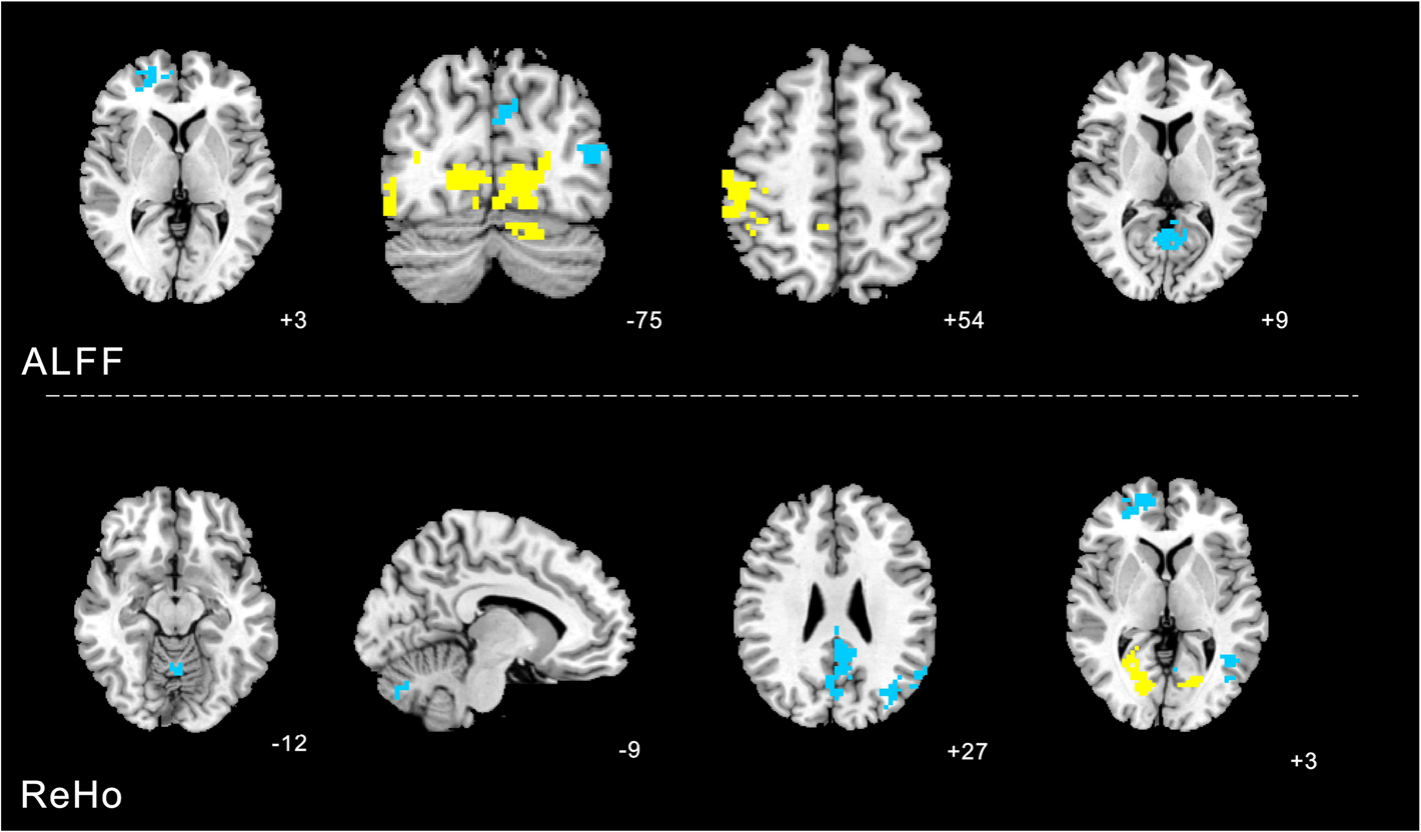
Changes of ALFF and REHO values before and after acupuncture at non-acupoint were calculated with paired *t*-test. The yellow color respects regions with higher ALFF and REHO values after real acupuncture at KI3 than before. The blue color respects in contrast

Increased ReHo was detected in right occipital lobe cuneus (BA18). Decreased ReHo was observed in the left cerebellum posterior lobe, left pyramis, cerebellum anterior lobe culmen, right medial frontal gyrus (BA10), left middle temporal gyrus (BA39) and left limbic lobe cingulate gyrus(BA31) (Table 3, Fig. 6).

**Table 3:** Changes of ALFF of cerebral area before and after acupuncture at non-acupoint

Cluster	Voxels	Brain areas	Brodmann area (BA)	Peak MNI coordinate			Peak intensity
				X	Y	Z	
ALFF	480	Left occipital lobe lingual gyrus	18	−12	−75	0	4.6955
	87	Right middle occipital gyrus	19	51	−75	−12	4.4098
	126	Right superior frontal gyrus	10	24	48	3	−3.9934
	102	Right middle temporal gyrus	39	−45	−75	12	−4.0215
	249	Right limbic lobe posterior cingulate	30	3	−63	9	−4.1024
	367	Right parietal lobe postcentral gyrus	40	48	−30	54	5.9415
REHO	122	Left cerebellum posterior lobe pyramis	-	−9	−75	−33	−4.7741
	107	Left cerebellum anterior lobe culmen	-	0	−60	−12	−4.9814
	90	Right medial frontal gyrus	10	15	54	3	−3.936
	85	Right occipital lobe cuneus	18	24	−54	3	4.1131
	205	Left middle temporal gyrus	39	−36	−72	27	−5.4515
	402	Left limbic lobe cingulate gyrus	31	−6	−45	27	−5.1418

### The difference of ALFF and ReHo changes between the KI3 and non-acupoint group

#### ALFF analysis

Increased ALFF was detected in the left cerebellum posterior lobe, left middle frontal gyrus (BA10), right superior frontal gyrus (BA10), left middle temporal gyrus (BA39) and right limbic lobe posterior cingulate (BA31). Decreased ALFF was observed in the left cerebrum occipital lobe lingual gyrus (BA18), right middle occipital gyrus (BA19) and parietal lobe postcentral gyrus (BA40).

#### ReHo analysis

**Fig. 7:**
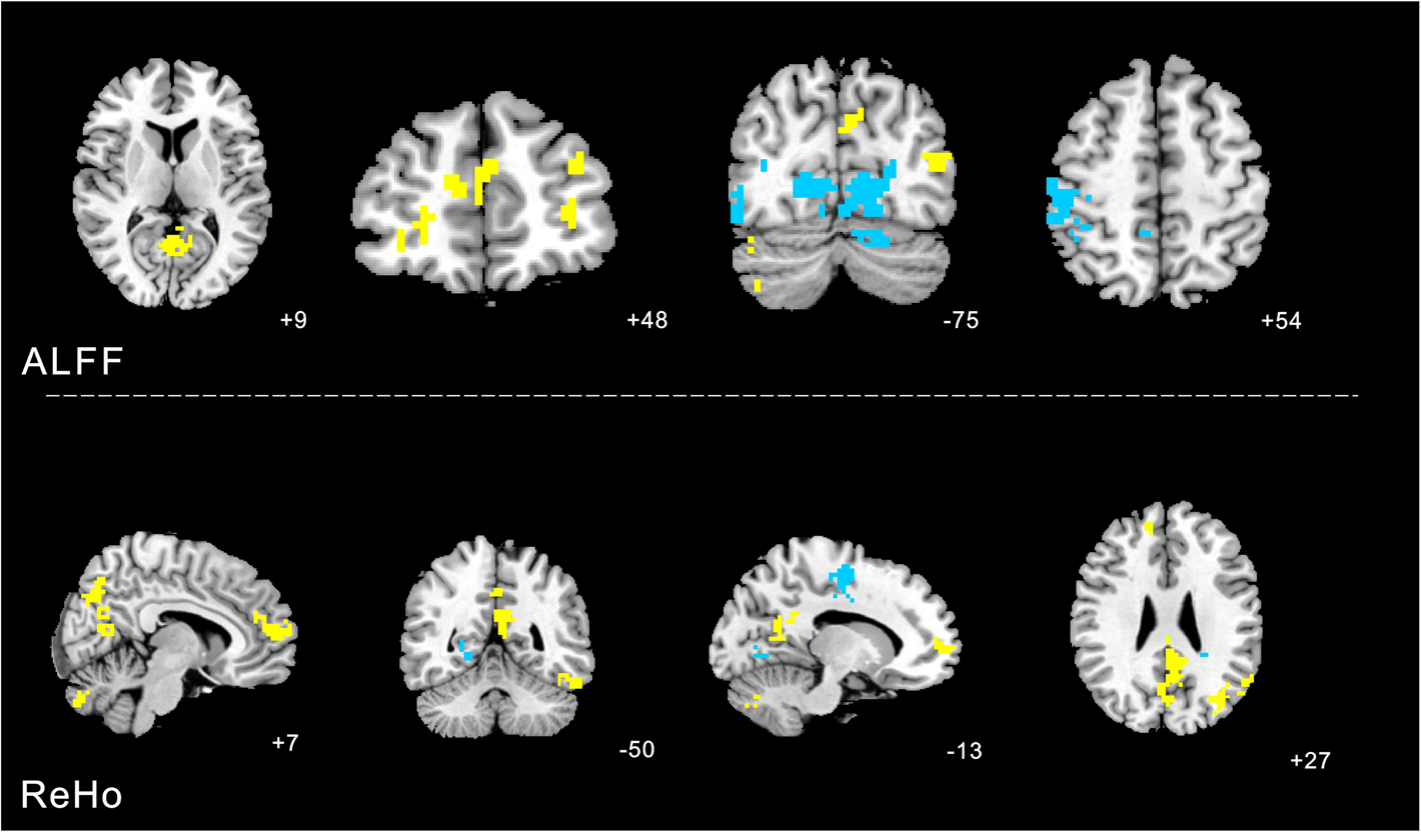
The difference of ALFF and ReHo changes between the KI3 and non-acupoint group was calculated. The yellow color indicates the changes in KI3 group show higher ALFF and ReHo compared with the changes in non-acupoint group. The blue color respects in contrast

Increased ReHo was detected in left cerebellum posterior lobe pyramis, left temporal lobe fusiform gyrus (BA37), left cerebellum anterior lobe, right medial frontal gyrus (BA10), left middle temporal gyrus (BA39) and left limbic lobecingulate gyrus (BA31). Decreased ReHo was observed in the right occipital lobe cuneus (BA18), limbic lobe cingulate gyrus (BA31) (Table 4, Fig. 7).

**Table 4:** The difference of ALFF and ReHo changes between the KI3 and non-acupoint group

	Voxels	Brain areas	Brodmann area (BA)	Peak MNI coordinate			Peak intensity
				X	Y	Z	
ALFF	88	Left cerebellum posterior lobe	-	42	−72	−45	4.1072
	480	Left cerebrum occipital lobe lingual gyrus	18	−12	−75	0	−4.6955
	87	Right middle occipital gyrus	19	51	−75	12	−4.4098
	109	Left middle frontal gyrus	10	−33	45	21	4.437
	126	Right superior frontal gyrus	10	24	48	3	3.9934
	102	Left middle temporal gyrus	39	−45	−75	12	4.0215
	249	Right limbic lobe posterior cingulate	31	3	−63	9	4.1024
	367	Right parietal lobe postcentral gyrus	40	48	−30	54	−5.9415
REHO	122	Left cerebellum posterior lobe pyramis	-	−9	−75	−33	4.7741
	134	Left temporal lobe fusiform gyrus	37	−45	−48	−21	4.7063
	107	Left cerebellum anterior lobe	-	0	−60	−12	4.9814
	218	Right medial frontal gyrus	10	3	60	12	6.5767
	85	Right occipital lobe cuneus	18	24	−54	3	−4.1131
	205	Left middle temporal gyrus	39	−36	−72	27	5.4515
	402	Left limbic lobecingulate gyrus	31	−6	−45	27	5.1418
	152	Left limbic lobe cingulate gyrus	31	−18	−21	39	−7.4947

## Discussion

Acupuncture at KI3 is usually used in the treatment of developmental retardation, presenility, diseases of the urogenital system, headache, dizziness, auditory disorders, sore throat, toothache, cognitive dysfunctions, insomnia, chronic cough, and chronic diarrhea according to traditional Chinese medicine, which has been proved by numerous studies and clinical trials [32–34]. Wang et al. [32] proved through clinical trials that acupuncture at KI3 positively regulated glucose and lipid metabolism in patients with Type 2 Diabetes Mellitus. Feng et al. [33] used fMRI-connectivity analysis and concluded that correlations between memory-related brain regions were enhanced following acupuncture at KI3 in patients with mild cognitive impairment. Takayama et al. [34] investigated the effects of acupuncture at KI3 and other points on retrobulbar circulation in patients with open-angle glaucoma, and concluded that acupuncture could improve the retrobulbar circulation and intraocular pressure.

Some researchers have proposed that specificity of acupoints has a close relationship with cortical functional activity. The brain receives signals generated at the acupoint as well as those related to diseases, does comprehensive analysis, and then sends signals to target organs for further regulation [33, 35].

When people undergo acupoint needling, they have a prompt response to the stimulation, and after a certain time, they begin to experience the effect of the needling. Hence, we focused our observation on the time period following the needling to understand the true effect of acupuncture. Mayer [36] investigated several clinical observations and acupuncture-analgesia studies and found that a delayed response exists following longer (>20 min) acupuncture stimulation. Moreover, Napadow et al. [37] used brainstem-focused cardiac-gated fMRI to evaluate time-variant brain responses to longer duration (>30 min) acupuncture stimulation, finding that electro-stimulation at acupoint ST36 produced linearly time-variant activity in limbic regions (amygdala, hippocampus, and substantia nigra), which was bimodal and not likely habituation-consisting of activation in early blocks, and deactivation by the end of the run. By finding higher changes in the number and intensity of activated/deactivated regions at 15 min after needle removal than at 5 min, Zheng et al. [38] also demonstrated lasting and strong after-effects of electroacupuncture on cortical function.

Based on the analysis of both of ReHo and ALFF values, acupuncture at KI3 had a relatively brain functional effect on BA7 compared with sham acupuncture, and a relatively centralized effect on BA6, BA7, BA10, BA20, and the posterior cerebellar lobe compared with non-acupoint stimulation. The cortical regions involved here are known to have a close relationship with motor planning and execution, integration of sensory inputs, and integration of visual information with prefrontal associations, and the posterior cerebellar lobe mainly affects starting, planning, and coordination of movement, including the determination of strength, direction and scope [39–43].

Thus, the fMRI results support our hypotheses to a certain degree. Motor planning and execution, sensory input, and visual information processing were partly coordinated in the clinical application of acupuncture at KI3. Chen et al. [44] used fMRI data and a multivariate granger-causality analysis of the effects of acupuncture at KI3 in patients with mild cognitive impairment and demonstrated the relative functional specificity of acupuncture at KI3. Feng et al. [33] also investigated acupuncture at KI3 in mild cognitive impairment. They used fMRI data to perform connectivity analysis using a whole-brain network and reached a similar conclusion. Moreover, Zhou Y, et al. [45] used fMRI to investigate the effect of acupuncture at KI3 and other acupoints on brain activity in patients with Alzheimer’s disease and concluded that acupuncture potentially affects Alzheimer’s disease, as there were right main hemisphere activations (temporal lobe, such as hippocampal gyrus, insula, and some area of parietal lobe) and left activated regions (temporal lobe, parietal lobule, some regions of cerebellum) impaired areas in brain for AD patients activated/deactivated. Unlike these experimental studies, we chose to analyze ReHo and ALFF values to quantify the effect of acupuncture at KI3, and set up the sham acupuncture and non-acupoint. This allowed us to use fMRI data to investigate healthy individuals and avoided some interference from uncertain variables and factors.

However, the sample number in this study was limited and the observations only focused on healthy volunteers. In the future we need to increase the sample size and try to analyze the specificity of KI3 under pathologic conditions.

## Conclusions

We used analyses of ReHo and ALFF values to demonstrate that compared with sham acupuncture and non-acupoint acupuncture, brain regions associated with visual and auditory perception, body movement, and association are selectively activated after acupuncture at KI3. We speculate that acupuncture at KI3 acts on certain brain regions related to its clinical application.

## Consent

Written informed patient consent was obtained for this study.

## Abbreviations

fMRI: Functional magnetic resonance imaging
ALFF: Amplitude of low-frequency fluctuation
ReHo: Regional homogeneity
BA: Brodmann area
PSM: Propagated sensation along meridians
TEAS: Transcutaneous electrical acupoint stimulation
EA: Electroacupuncture
DPARSF: Data Processing Assistant for Resting-State fMRI
SPM8: Statistical Parametric Mapping
REST: Resting-State fMRI Data Analysis Toolkit
MNI: Montreal Neurological Institute
KCC: Kendall’s coefficient of concordance
